# mRNA-Based Personalized Cancer Vaccines: Opportunities, Challenges and Outcomes

**DOI:** 10.32607/actanaturae.27707

**Published:** 2025

**Authors:** A. A. Ibragimova, A. A. Fedorov, K. M. Kirilenko, E. L. Choynzonov, E. V. Denisov, M. R. Patysheva

**Affiliations:** Cancer Research Institute, Tomsk National Research Medical Center, Russian Academy of Sciences, Tomsk, 634009 Russia; Center for Systems Bioinformatics, Tomsk National Research Medical Center, Russian Academy of Sciences, Tomsk, 634050 Russia

**Keywords:** mRNA vaccine, cancer, immunotherapy, neoantigens, liposomes, clinical trials

## Abstract

mRNA-based cancer vaccines represent an innovative approach to cancer treatment. Cancer mRNA vaccines are structurally based on specific tumor antigens, a technique which enables the patient’s immune system to become activated against cancer cells. Clinical trials of mRNA vaccines against various types of tumors, including melanoma, lung cancer, pancreatic carcinoma, breast cancer and others, are currently underway. Because of their favorable safety profile and adaptability, these therapeutics hold considerable promise in efforts to enhance cancer treatment efficacy and prolong patient life. This review outlines steps in the development of manufacturing technologies for mRNA-based therapeutics, describes the algorithm used to design personalized anti-tumor mRNA vaccines, discusses their practical implementation, and summarizes current clinical trials in cancer immunotherapy.

## INTRODUCTION

Cancer is a leading cause of death and disability worldwide, which justifies its status as a top medical and societal concern. Despite decades of innovation, solid tumors remain among the leading causes of cancer-related mortality worldwide, owing to their high incidence and the complexity of achieving effective intervention [1]. Even with refined treatment protocols, long-term survival remains hard to achieve: in lung cancer – the most frequently diagnosed cancer – more than 50% of patients do not survive beyond 3.5 years post-diagnosis [2].

Novel therapeutic strategies are urgently needed to enhance treatment efficacy and improve both survival and the quality of life of cancer patients. In this regard, modulation of the anti-tumor immune response holds particular promise. The inclusion of immunotherapy with immune checkpoint inhibitors in clinical guidelines has significantly improved treatment efficacy with melanoma, lung cancer, breast cancer, ovarian cancer, and other types of solid tumors [3, 4]. Nucleic-acid–based anti-tumor vaccines, particularly those utilizing DNA or mRNA platforms, represent a promising frontier in cancer immunotherapy

**Fig. 1:**
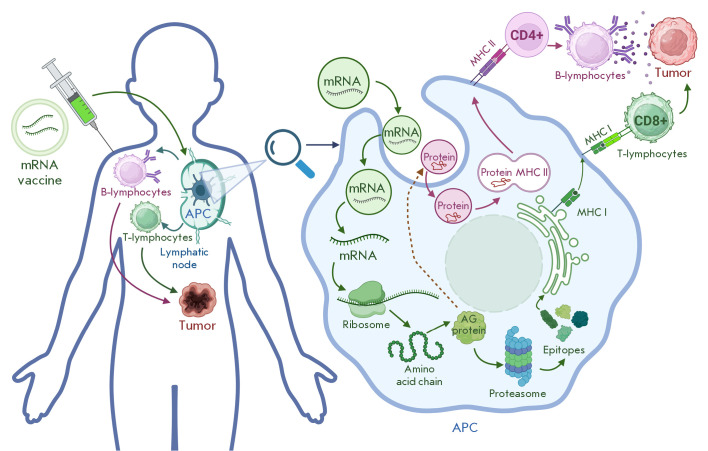
Anti-tumor mRNA vaccine mechanism. mRNA – messenger ribonucleic acid, APCs – antigen-presenting cells, AG protein – antigenic protein

mRNA-based anti-tumor vaccines exploit the natural protein synthesis machinery of antigen-presenting cells (APCs): by delivering transcripts encoding tumor antigens into the cytoplasm, mRNA enables endogenous production and immunogenic presentation of the target antigen. Following processing, proteins associated with the target antigen (epitopes) can appear on the surface of APCs by binding to the molecules of the major histocompatibility complex classes I and II – MHC I and MHC II, respectively (Fig. 1). The resulting immune activation engages both of the arms of adaptive immunity: CD4^+^ T helper cells and B cells (for antibody production), as well as CD8^+^ cytotoxic T lymphocytes, which are capable of directly eliminating target cells [5]. mRNA-based vaccine platforms offer advantages such as:

1) Enhanced stability and translational efficiency. Advances in nucleotide modification and delivery technologies have rendered mRNA more resistant to degradation and significantly improved its protein expression in target cells [6]

2) Intrinsic immunostimulatory properties. The mRNA molecule itself can activate the innate immune system, thereby acting as a built-in adjuvant that enhances vaccine efficacy [7].

3) Favorable safety profile. Unlike DNA vaccines or viral vectors, mRNA remains extranuclear and does not integrate into the host genome, thereby eliminating the risk of insertional mutagenesis [8].

4) Economical and scalable production pipeline. The development of personalized mRNA vaccines relies on the synthesis of a single DNA template, followed by enzymatic in vitro transcription to yield large quantities of mRNA – a streamlined process that is substantially less resource-intensive than the complex manufacturing required for viral vector or plasmid DNA vaccines.

This review critically assesses the promise of mRNA-based therapeutic vaccines in solid malignancies, addressing key aspects, including mRNA design and production, delivery systems for efficient targeting of APCs, and the status of ongoing and completed clinical trials.

## KEY MILESTONES IN THE EVOLUTION OF mRNA TECHNOLOGIES

**Fig. 2:**
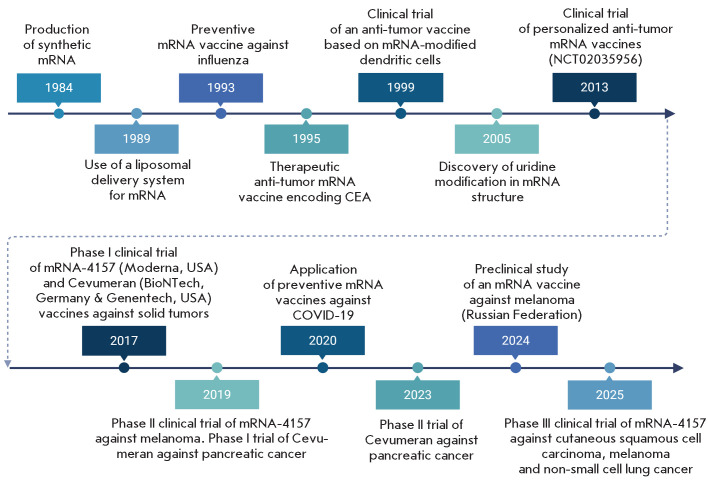
Development history of mRNA-based vaccine production and application technologies. CEA – carcinoembryonic antigen, DC – dendritic cells, mRNA – messenger ribonucleic acid

Despite the discovery of mRNA and transcription in the 1960s, the therapeutic potential of synthetic mRNA was not immediately understood. A pivotal shift occurred in 1984, when researchers demonstrated that in vitro–transcribed mRNA could direct functional protein expression in cells, laying the foundation for mRNA-based gene regulation and therapy [9] (Fig. 2). Early progress in mRNA therapeutics was hampered by the molecule’s susceptibility to degradation and inefficient cellular delivery [10]. This challenge was first overcome in 1989, with the successful delivery of synthetic Photinus pyralis luciferase mRNA into murine cells via liposomes formulated with the cationic lipid DOTMA (N-[1-(2,3-dioleoyloxy) propyl]-N,N,N-trimethylammonium chloride) [11]. In the 1990s, most companies that had pursued mRNA vaccine development redirected their investments elsewhere, as the production of stable liposomal mRNA formulations remained prohibitively expensive. Nevertheless, research continued: as early as 1990, a landmark study demonstrated that synthetic mRNA could be expressed in vivo following direct injection into mice [12, 13]. In 1993, researchers synthesized the first prophylactic mRNA vaccine, designed to express the nucleoprotein of the influenza virus and demonstrated its ability to activate antigen-specific cytotoxic T lymphocytes in murine models [14].

he first evidence of anti-tumor immunity induced by mRNA vaccination was reported in 1995, following intramuscular delivery of a mRNA-encoding carcinoembryonic antigen (CEA) into mice [15]. Subsequently, in 1999, using a mouse melanoma model, it was demonstrated that the introduction of gp100 mRNA, which encodes the melanosome matrix glycoprotein, into the spleen inhibits tumor growth [16]. Meanwhile, a Phase 1 clinical trial was initiated to activate antigen presentation in autologous dendritic cells from prostate cancer patients by means of synthetic mRNA encoding prostate-specific antigen (PSA) [17]. In 2000, Ingmar Hoerr et al. discovered that direct injections of mRNA can induce an immune response in mice and, then, with the promising development of mRNA vaccines in mind, CureVac (Germany) was incorporated, a company that remains one of the leading developers of mRNA-based vaccines to this day [18, 19].

he seminal work of Katalin Karikó and Drew Weissman laid the groundwork for modern mRNA therapeutics. During early efforts to develop an mRNA-based HIV vaccine in the late 1990s, they discovered that unmodified mRNA activated innate immune pathways – specifically through Toll-like receptors (TLR3, TLR7, TLR8) – eliciting a robust inflammatory response in murine models [20]. A pivotal advance happened in 2005, when Karikó and Weissman reported and patented the incorporation of pseudouridine, in place of uridine, within mRNA. This chemical modification prevented recognition by innate immune sensors, thereby suppressing inflammatory responses and markedly improving translational efficiency – a discovery that underpins the development of modern mRNA vaccines [21, 22]. In 2023, Katalin Karikó and Drew Weissman were awarded the Nobel Prize in Physiology or Medicine for their discovery that nucleoside-modified mRNA can suppress innate immune activation – a breakthrough that enabled the development of effective mRNA vaccines [23].

Improvements in mRNA-based technology have enabled pharmaceutical companies such as Moderna and Pfizer-BioNTech to develop effective mRNA vaccines against COVID-19 [6]. The successful and expanded clinical use of mRNA vaccines has driven the rapid advancement and optimization of the entire mRNA manufacturing pipeline [24]. Moreover, mRNA technologies are suitable for creating preparations not only against infectious diseases (rabies, influenza, Epstein-Barr virus, Zika virus, Nipah virus, etc.), but also against oncological diseases, such as prostate cancer, hepatocellular carcinoma, melanoma, and non-small cell lung cancer, thereby attracting the attention of scientists and biotechnology and pharmaceutical companies in Russia, the United States, Germany, China, and other countries [24]. Against this background, the first clinical trial of a personalized mRNA-based vaccine against melanoma (NCT02035956) was initiated in 2013 [25].

zed therapy represents the most promising strategy in modern oncology. mRNA-based anti-tumor vaccines targeting tumor neoantigens – unique antigens arising from somatic mutations in malignant cells – have demonstrated high efficacy. Neoantigens are broadly classified into two categories: shared (or common) neoantigens, which occur across multiple patients and are absent from the normal genome, and personalized (or private) neoantigens, which are unique to an individual’s tumor mutanome [26, 27]. Shared neoantigens represent promising targets for “off-the-shelf” therapeutic cancer vaccines with broad applicability, whereas personalized neoantigens – though patient-specific – have demonstrated remarkable therapeutic efficacy in clinical settings [28, 29, 30, 31, 32]. Production of a personalized anti-tumor mRNA vaccine involves a sequential workflow: (1) comprehensive profiling of the patient’s tumor neoantigen repertoire, (2) computational design of the mRNA construct, (3) synthesis of the DNA template, (4) in vitro transcription to generate mRNA, and (5) formulation into a delivery vehicle, such as lipid nanoparticles.

**Identification of tumor neoantigens**

The identification of neoantigens, defined as patient-unique tumor antigens generated by somatic mutations, represents the cornerstone of personalized mRNA vaccine design. This process involves a multimodal genomic analysis including whole-exome sequencing (WES), whole-genome sequencing (WGS), and transcriptome profiling, coupled with advanced computational algorithms to predict and rank neoantigens based on immunogenicity and expression levels [33, 34]. At the same time, DNA sequencing makes it possible to identify somatic mutations (missense, nonsense, deletions, insertions, etc.) that potentially encode neoepitopes, while RNA sequencing confirms their expression status, which serves as an important criterion for selecting neoantigens [35]. Additionally, the use of RNA sequencing allows for the false positives detected in a DNA sequencing analysis but not actually expressed to be excluded [35]. Actually, comparing DNA and RNA sequencing data in practice yields more reliable results when forming a pool of potential neoantigens [34].

Once the “raw” data has been collected, it undergoes preliminary processing, including quality control (using FastQC1 ), filtering and trimming of incorrect sections (Trimmomatic or Cutadapt), and alignment of reads to the reference genome (Bowtie 2) [36, 37, 38]. The subsequent step involves identifying somatic mutations in the tumor as compared to normal samples, using tools such as MuTect2 (from the GATK pipeline), Strelka, or VarScan2 [39, 40, 41]. In addition, the variant allele frequency (VAF) is calculated, reflecting the proportion of mutations in the tumor cell genome [42]. Simultaneously, RNA sequencing data is analyzed using STAR + RSEM, the Salmon or Kallisto pipeline which allows quantitative expression metrics to be collected – TPM (Transcripts Per Million) and FPKM (Fragments Per Kilobase of transcript per Million mapped reads) [43, 44, 45, 46]. Such normalization approaches incorporate both the transcript length and sequencing depth, allowing for reliable cross-sample and cross-transcript expression quantification, which is essential in prioritizing immunogenic neoantigens [34].

The next stage involves running computational predictions of neoepitopes and an assessment of the likelihood that they would elicit a T-cell-mediated immune response. Determination of the patient’s HLA genotype using, for example, the OptiType algorithm is particularly significant [47]. The binding affinity of mutant peptides to MHC I/II molecules is also assessed using various tools, the most popular of which are NetMHC and NetMHCpan, MHCflurry, and IEDB [48, 49, 50]. With these tools, the IC_50_, or percentile rank, is calculated, allowing epitopes with a high predicted binding affinity (IC_50_ < 500 nM) to be sampled. Today’s neoantigen prioritization strategies incorporate multiple biological and computational parameters: the expression level of the mutant allele, variant allele frequency (VAF), dissimilarity of the mutant peptide from its wild-type counterpart, and the thermodynamic stability of the peptide-MHC complex [34]. Although in silico neoantigen screening is standard in personalized mRNA vaccine pipelines because of its efficiency, immunopeptidomics-mass spectrometrybased identification of naturally presented peptide– MHC complexes offers definitive validation of surface presentation [51, 52].

While predicting which neoepitopes will elicit a strong immune response is far from a perfect approach, the synergy of multi-omics data and intelligent computational models now offers a powerful and increasingly reliable strategy for designing personalized mRNA vaccines with real therapeutic potential [34].

**Key structural elements of mRNA**

**Fig. 3:**
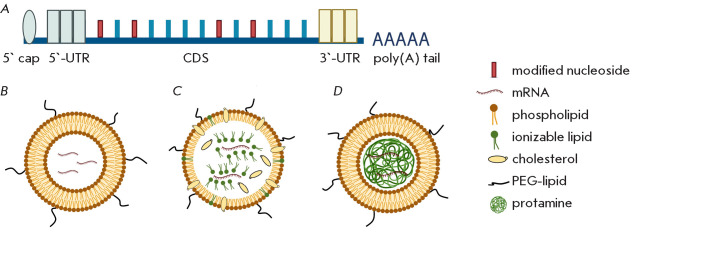
Structural components of mRNA vaccine. (A) – mRNA molecule composition; (B) – liposome structure; (C) – lipid nanoparticle structure; (D) – lipoliplex structure. 5’ cap – cap, 5’-UTR and 3’-UTR – untranslated regions, CDS – coding sequence

Modern mRNA vaccines are engineered with an optimized molecular architecture to enhance stability, maximize protein expression, and minimize unintended immune activation [53]. The mRNA molecule has several essential elements (5’-cap, 5’-UTR, coding sequence, 3’-UTR, and poly(A)-tail), each of which plays a key role (Fig. 3A) [53].

The rational engineering of therapeutic mRNA now relies heavily on advanced bioinformatic software capable of predicting higher order RNA structures. Secondary and tertiary folding patterns – key determinants of mRNA stability, innate immune activation, and protein yield in APCs – are modeled using tools such as RNAfold, NUPACK, and mfold. These platforms facilitate the identification of structurally optimal regions where to incorporate modified nucleosides, thereby fine-tuning vaccine efficacy and safety [54, 55, 56].

The 5′ Cap is the most critical structural element of mRNA, as it protects the transcript from exonucleolytic degradation and facilitates the initiation of translation (Fig. 3A). Several types of caps are classified: Cap0, Cap1, Cap2, m_6_Am Cap. Modern technologies, such as CleanCap, enable a capping efficiency of up to 99%, which is critical for the synthesis of target proteins in APCs [53]. Modified nucleosides (pseudouridine, 5-methylcytidine, N1-methylpseudouridine) are often involved in mRNA production, which increases expression levels and reduces innate immunogenicity. The cap is followed by a 5’-untranslated region (5’-UTR) which affects the stability and efficiency of translation.

The coding sequence (CDS), located in the central part of the molecule, contains information about the target antigen. In mRNA-based antitumor vaccines, these may be tumor-associated antigens (TAAs) or tumor-specific antigens (TSAs). Multiple antigens can be encoded simultaneously, which enhances the immune response [57, 58]. Codons optimization improves the speed and accuracy of translation, thereby enhancing vaccine efficacy [57]. The 3′ end of mRNA comprises the 3′-untranslated region (3′-UTR) and a polyadenylated tail, which together modulate mRNA decay kinetics, subcellular localization, and translational persistence (Fig. 3A) [53].

In addition to linear mRNA molecules, self-replicating mRNAs are being developed that include viral replication elements which increase their copy number in cells and thereby reduce the required dose of the mRNA preparation [53]. Circular mRNAs with a closed structure are an alternative, allowing mRNAs to remain in the body over a longer period of time and ensuring more prolonged antigen expression [58]. Both areas are being actively researched, with account of the potential to improve the efficacy and safety of mRNA-based vaccines [53, 58].

**Delivery systems for mRNA-based cancer vaccines**

The mRNA molecule that has been administered to the patient must be delivered to the APCs without it losing its integrity. Selecting the optimal mRNA delivery system is an important step in the production of mRNA-based vaccines. The most commonly used mRNA-based delivery systems include lipid platforms, which comprise liposomes, lipid nanoparticles (LNPs), and lipopolyplexes (LPPs). They all differ in structure and functional characteristics (Fig. 3B–D).

Liposomes consist of a bilipid layer forming an outer shell inside which mRNA is encapsulated. The surface of liposomes may contain polyethylene glycol (PEG) molecules, which provide steric stabilization and increase circulation time in the blood. LNPs are more complex optimized structures that include ionizable lipids, phospholipids, cholesterol, and PEG-lipids, which not only effectively encapsulate mRNA but also protect it from degradation and ensure that it is delivered into the cells’ cytoplasm [59]. LNPs are successfully utilized in antitumor mRNA-based vaccines, in which they demonstrate high stability and delivery efficiency [53, 60].

The efficiency of lipid delivery platforms is affected by a variety of factors, such as size, charge, lipid composition, membrane phase state, antigen localization method, and the presence of immunomodulatory components.

The size of mRNA delivery vehicles dictates their biodistribution and the immunological outcome. Small nanoparticles – typically ≤ 100 nm for lipid nanoparticles (LNPs) and < 200 nm for conventional liposomes – readily access lymphoid tissues, engage resident dendritic cells, and are associated with Th2-polarized responses. In contrast, larger particles ( > 100 nm for LNPs; > 500 nm for liposomes) exhibit prolonged retention at the injection site, creating an antigen deposit that supports Th1-type immunity [60, 61, 62, 63].

Particle charge also plays a significant role: cationic particles, for example, based on dioctadecyl dimethylammonium bromide (DDA), are actively absorbed by APCs, promote cross-presentation and the activation of CD8^+^ T-lymphocytes, whereas neutral and anionic liposomes predominantly induce humoral immunity [64, 65]. In LNPs, ionizable lipids acquire a positive charge at low pH values in endosomes, facilitating the release of mRNA into the cytoplasm [66].

The phase state of the bilipid layer determines the ability of liposomes to fuse with cell membranes and release antigen intracellularly. Liquid-crystalline liposomes facilitate cross-presentation via MHC I, whereas more rigid liposomes induce a pronounced Th1 response in vivo [66, 67, 68]. Cholesterol, which is part of the membrane, increases the stability of liposomes and may enhance or reduce complement activation depending on its charge and size [66, 69].

The addition of immunomodulatory components such as Toll-like receptor ligands, e.g. CpG-oligodeoxynucleotides (CpG-ODNs), poly(I:C), synthetic glycolipids and cytokines, allows the immune response to be directed towards the desired type of inflammation. Specifically, CpG-ODNs recognized by TLR9 and trehalose-6,6’-dibeheneate (TDB) promote the induction of a Th1 response accompanied by IFN-γ production [70, 71, 72, 73]. Poly(I:C), which mimics viral double-stranded RNA and activates TLR3, enhances the cross-presentation of antigen and stimulates the development of a cytotoxic T-lymphocyte response [74, 75]. Additionally, the combination of TDB with lipids such as DDA may lead to the activation of the Th17 response and the production of IL-17 [67, 76, 77, 78].

Encapsulated antigens have been demonstrated to efficiently enter the intracellular compartments of APCs, where they are processed and presented via both class I and class II MHC molecules, enabling the activation of CD8^+^ and CD4^+^ T-lymphocytes [68]. At the same time, antigens associated with the surface of liposomes have a lower capacity for intracellular processing but may be accessible for direct recognition by B-lymphocytes via BCR receptors, contributing to the formation of a humoral response [68, 79].

Lipopolyplexes (LPPs) are hybrid systems that combine cationic lipids, such as DOTAP (1,2-dioleoyl-3-trimethylammonium-propane), and polymers, such as protamine, to form stable complexes with mRNA [80]. Lipids protect mRNA and facilitate its delivery across cell membranes, while polymers enhance mRNA compaction, increasing the stability of the complex. LPPs are highly stable in vitro and effectively deliver mRNA, including self-replicating mRNA, to dendritic cells, eliciting a strong immune response. LPPs used to deliver mRNA encoding neoantigens have been demonstrated to induce potent T-cell responses and exhibit anti-tumor activity in mouse models [80].

Beyond lipid-based systems, alternative mRNA delivery strategies for targeting APCs include polymeric nanoparticles, dendrimers, peptide-based complexes, physical methods such as jet injection and electroporation, and engineered viral vectors.

Polymeric nanoparticles, such as poly(β-aminoesters), are biodegradable polymers containing amino and ether groups in their structure, which enables them to bind mRNA through electrostatic interactions. The flexibility in modifying polymer nanoparticles provides the ability to vary the molecular weight, degree of branching, and polymer chemical composition, optimizing the charge, particle size, and their ability to protect mRNA from enzymatic degradation [53].

Dendrimers are highly branched polymer molecules with a tree-like structure. Dendrimers feature a compact central core – typically a small molecule or ion – serving as the focal point for the iterative, layer-by-layer growth of branched monomeric units, resulting in a well-defined, tree-like nanostructure. Functional groups such as amines or hydroxyl groups are located on the outer surface of the dendrimer, which confers the ability to bind and protect mRNA [53]. Peptide complexes consist of mRNA bound to cationic peptides such as protamine, which form dense nanoparticles as a result of electrostatic interactions between positively charged peptides and negatively charged mRNA, protecting it from degradation and facilitating its penetration into cells [53].

Jet injection allows researchers to deliver “naked” mRNA without carriers using jet injectors such as PharmaJet or Bioject. The devices have no needles and use high pressure (up to 1,000 bar) to push mRNA through the skin into the subcutaneous fat or muscle tissue. An mRNA penetration mechanism into cells is based on a temporary disruption of cell membrane integrity as a result of mechanical stress caused by a high-speed jet, which allows mRNA to reach the cytoplasm of APCs [81]. Studies show that introducing mRNA using this method can trigger an innate immune response comparable to that induced by LNPs [81].

Electroporation is primarily used for ex vivo delivery of mRNA into dendritic cells or other immune cells which are subsequently administered to the body. This method involves the use of electrical pulses to temporarily increase the permeability of cell membranes, facilitating penetration by the mRNA. Electroporation is effective for activating the immune response, but its use in vivo remains limited as a consequence of the risk of tissue damage and the complexity of implementation [53].

Viral vectors, more commonly used to deliver selfreplicating mRNA, consist of a modified viral genome containing mRNA or self-replicating mRNA, as well as a protein capsid or lipid envelope that enables cell penetration. Adenoviruses, lentiviruses, or alphaviruses modified to express tumor antigens are often used. Self-replicating mRNA includes viral replication elements that enhance antigen translation in cells, reducing the required dose of the mRNA vaccine and enhancing the immune response. Vaccines utilizing viral vectors are being actively explored as immunotherapeutic agents against multiple cancer types, with particularly promising results in preclinical studies with HPV-driven tumors [82].

**Storage and transportation of mRNA-based therapeutics**

Immunobiological medicinal preparations, which include all known vaccines, are stored at a temperature between +2°C and +8°C1 . The exception is mRNA-based vaccines, which are classified as biotechnology-derived medicinal preparations (BDMPs) with specific storage and transportation requirements2 . As an example, Pfizer’s mRNA vaccine is stable for 6 months at −80°C and only 5 days at +2 to +8°C. Moderna’s vaccine can be stored for 6 months at −20°C but 30 days at 2–8°C [83].

Storage and transportation of mRNA vaccines requires strict temperature control as specified by the manufacturer and special equipment such as refrigerators and freezers, refrigerated boxes, and vaccine carriers that can be stored at −80°C and meet the performance standards as defined by the World Health Organization (WHO) [84].

One of the newest methods of delivery of mRNA into a patient’s body is the use of micro-needle chips [84]. This method allows the mRNA preparation to be stored and transported at room temperature for several months. To date, this method has been applied exclusively to anti-infective mRNA vaccines; the technical intricacies and scalability limitations of chipbased production systems render it poorly suited to personalized cancer vaccine development.

All of the above-mentioned transportation issues lead to certain difficulties in the further implementation of mRNA vaccines; however, they do not make their use impossible in clinical practice. One way to resolve this issue could be to manufacture and use the preparation within a single institution, which is currently being done in the Russian Federation through Resolution No. 213 dated February 24, 2025, related to BDMPs intended for use in accordance with individual medical prescriptions.

**Administration strategies for therapeutic mRNA cancer vaccines**

Selecting an appropriate route of administration is essential in maximizing the therapeutic potential of mRNA vaccines while minimizing off-target effects and systemic toxicity. Administration routes have different characteristics and influence the distribution of the vaccine in the body, the type of immune cells activated, and, consequently, the strength and duration of the response. mRNA-based vaccines can be administered intradermally, subcutaneously, intranasally, intranodally, intraperitoneally, intramuscularly, and intravenously. In modern clinical trials, intravenous, intramuscular, and subcutaneous administration of mRNA vaccines are the most commonly practiced protocols.

In intravenous administration, the preparation penetrates the systemic bloodstream, spreading throughout organs and tissues, and rapidly reaches the APCs. This method allows for the administration of significant volumes of the vaccine and repeated runs to ensure a high level of anti-tumor immunity [58]. Data from clinical trials of BioNTech SE’s intravenous vaccine Cevumeran have confirmed its safety, good tolerability, and effectiveness in stimulating an immune response against cancer cells [32, 85]. This method of administration may, however, cause the development of a generalized febrile syndrome and flu-like symptoms, and there is also a risk of systemic toxicity, which is important to consider when planning studies. As a consequence of the specific structure of the liver’s vascular network and the mechanism of receptormediated uptake of mRNA vaccines by hepatocytes, these vaccines have an increased tropism for this organ, which can lead to immune-mediated hepatitis or hepatotoxicity [86]. With this method of preparation administration, it is essential to conduct a risk-benefit analysis of the treatment, and this puts restrictions on mRNA vaccine treatment.

Intramuscular administration of vaccines is better tolerated compared to intravenous administration. As a result of the muscle tissue’s good vascularization and the presence of APCs precursors that migrated during ontogenesis and converged to the injection site, intramuscular administration is sufficient to induce an anti-tumor immune response. The additional advantages of this method include flexibility in selecting the dose administered, the possibility of repeated administration to maintain anti-tumor immune activity, and a reduced risk of adverse reactions at the injection site [87]. The only side effects as relates to this method may be fever and flu-like symptoms, which can be treated with anti-inflammatory preparations. Moderna’s mRNA-4157 vaccine, encapsulated in lipid nanoparticles, was administered intramuscularly in all clinical trials, demonstrating sufficient safety and eliciting clinical responses in patients with melanoma and solid tumors [88, 89]. Considering its numerous advantages, the intramuscular route is widely used for the administration of already-approved anti-infective mRNA-based vaccines, including prophylactic preparations against SARS-CoV-2 [90, 91, 92, 93].

Intradermal and subcutaneous methods of mRNA administration may be implemented using either the traditional syringe method, microchips, or jet injection [81, 84]. Intradermal administration of mRNA-based vaccines stimulates a Th1-type immune response, which is explained by the high concentration of APCs in the dermis and epidermis layers and the favorable microenvironment for antigen transfer [94]. Whilst this method provides an opportunity to use smaller volumes of the preparation, it often leads to local adverse reactions, such as swelling, soreness, hyperemia, and itching [95, 96]. As opposed to this, the subcutaneous method of administration is characterized by a lower number of APCs in the subcutaneous adipose tissue, which requires an increase in dosage and the use of multiple injection sites. A slow absorption rate following subcutaneous administration, however, may contribute to mRNA degradation, reducing treatment efficacy [97]. Nevertheless, this route of administration has been used in mRNA-based vaccine trials before and is actively used in clinical trials conducted in China (NCT03908671, NCT05949775, NCT05761717) [80, 98, 99].

The intranasal route delivers the mRNA molecule to the APCs of peripheral lymphatic vessels, while the intranodal route delivers it to lymphatic APCs. At the same time, the implementation of these methods is complex and has limitations in terms of the volume of administered preparation [100]. The intraperitoneal method has similar limitations and is more commonly used to deliver mRNA-based vaccines encoding costimulatory immune molecules [101].

In selecting the administration method, the type of mRNA-based vaccine should be taken into consideration. As an example, to ensure the efficiency of native mRNA delivery in vivo, intradermal or intranodal methods are more commonly used due to the assumption that immature dendritic cells located in the dermis and lymph nodes are capable of absorbing mRNA through micro-pinocytosis [102].

Lipid nanoparticles, as a popular delivery tool, are compatible with virtually all known methods of administration. Intramuscular and intradermal administration, however, results in the longest mRNA transmission, with a half-life of more than 20 h, while intravenous administration results in a half-life of only 7 h [103]. A comparison of the anti-tumor effect and immunogenicity of intramuscular, intradermal, and subcutaneous administration of LPP-CT26 in CT26-luc mice with lung metastases was undertaken to evaluate the optimal method of vaccine delivery [80]. Mice that received the preparation subcutaneously had fewer metastatic lesions in the lungs, showed increased IFN-γ secretion, and greater anti-tumor efficacy when the number of injection sites was increased without a change in the dose, compared with the other two groups. This further illustrates the impact of optimizing the method of mRNA vaccine administration versus the anti-tumor response.

In summary, the method used to administer the mRNA-based vaccine is one of the key factors determining its efficacy and safety. All routes of administration have their advantages and disadvantages, which affect the distribution of the preparation in the body, the activation of immune cells and, as a result, the strength and duration of the immune response. Intravenous administration ensures systemic distribution, but it carries the risk of toxicity and high tropism for the liver. Intramuscular administration, due to its simplicity and safety, remains the most popular, providing flexibility in dosage and the possibility of repeated injections. Intradermal administration stimulates a potent Th1-type immune response but may cause local reactions. Subcutaneous administration, to the contrary, requires an increase in dosage due to slow absorption. Optimization of the method, dose, and frequency of administration, with consideration as to the type of mRNA-based vaccine and delivery system, is a prerequisite for achieving maximum anti-tumor efficacy and minimizing adverse reactions. Further studies in this area will enable the development of personalized vaccination strategies aimed at achieving a clinical response and minimizing adverse reactions.

## CURRENT STATUS OF CLINICAL TRIALS OF mRNA-BASED CANCER VACCINES

**Fig. 4:**
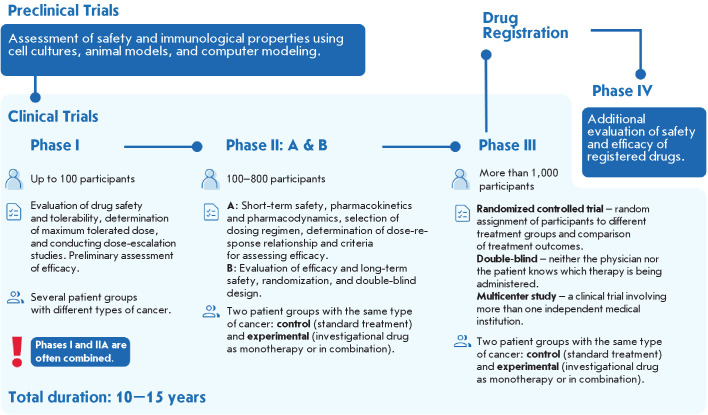
Anti-tumor mRNA vaccine trials stages required for preparation registration

Therapeutic mRNA vaccines targeting cancer are being developed globally, and most have transitioned successfully from preclinical validation into clinical evaluation (Table 1). Regulatory authorization as an oncology treatment requires the successful completion of three sequential clinical trial phases, with Phase III – focused on efficacy in large patient cohorts – representing the lengthiest and most complex stage (Fig. 4).

Preclinical trials of mRNA-based vaccines include an assessment of safety and immunological properties in vitro and/or ex vivo and in vivo in animal models. The success of preclinical trials opens an opportunity to proceed to Phase I clinical trials, where the therapeutic dose is determined and the preliminary efficacy of the preparation in treating patients is evaluated. This phase is usually undertaken on healthy volunteers, but in the case of anti-tumor mRNA-based vaccines, studies commence directly with target patients, resulting in the combination of phases I and IIa (NCT06307431, NCT06305767), as well as the emergence of dose escalation and expansion stages [104, 105]. Phase II can be divided into a pilot phase IIa, which evaluates short-term safety, establishes a dosing regimen, determines the dose-response relationship, and defines efficacy assessment criteria, and a more extensive controlled Phase IIb, which is necessary to determine the efficacy, safety, and optimal dosage of the preparation, as well as to make a decision about whether to proceed to the next phase. In the second stage, a comparison group receiving standard therapy is required. The most time-consuming and costly Phase III is a randomized, controlled, double-blind, multicenter study with a mandatory control group, which allows researchers to evaluate the efficacy and safety in a large number of patients. Successful completion of this stage leads to the preparation dossier being submitted to the authorized body for registration. In the case of personalized mRNA-based vaccines, it is not each specific preparation that is registered, but rather the technology used to produce it. Furthermore, in the Russian Federation, clinical trials of personalized anti-tumor mRNA-based vaccines related to BDMPs are permitted after their efficacy and safety have been proven and without the need for clinical studies.

**Table 1:** Clinical trials of personalized anti-tumor mRNA vaccines from 2021 to 2025

Drug name	Country, Sponsors	NCT ID	Phase	Status, Study Years	Tumor Localization	Treatment	Study Results
mRNA-4157 (V940	USA, Moderna, MSD	CT03313778 (KEYNOTE-603)	I	Open, recruiting 2017–2025	Any malignant tumors with MSI-H or other dMMR	Three groups: mRNA4157 monotherapy, combination mRNA-4157 + pembrolizumab, combination mRNA-4157 + pembrolizumab + chemotherapy.	eoantigen-specific T-cell responses were detected in all patients (n = 33); no adverse events of grade ≥3 were observed.
		NCT03897881 (KEYNOTE-942)	II	Open, recruiting 2019–202	igh-risk melanoma (stages IIIB–D or IV	Two groups: combination mRNA4157 + pembrolizumab, pembrolizumab monotherapy (control group	With a median follow-up of 18 months, recurrence-free survival was 79% in the combination therapy group (n = 107) versus 62% in the monotherapy group (n = 50).
		NCT06307431 (INTerpath-004)	I-II	Open, recruiting 2024–2032	Renal cell carcinoma after surgical resection	Three groups: mRNA4157 monotherapy, combination mRNA-4157 + pembrolizumab, pembrolizumab monotherapy (control group).	No results posted
		NCT06305767 (INTerpath-005)	I-II	Open, recruiting 2024–203	Muscle-invasive urothelial carcinoma after surgical resect	Three groups: combination mRNA4157 + pembrolizumab, pembrolizumab monotherapy (control group), combination mRNA-4157 + pembrolizumab + enfortumab vedotin + surger	No results posted
		NCT06295809 (INTerpath-007)	III	Open, recruiting 2024–203	table locally advanced operable cutaneous squamous cell carcinoma	Three groups: combination mRNA-4157 + pembrolizumab + surgery, surgery + radiotherapy, pembrolizumab monotherapy + sur	No results posted
		CT05933577 (INTerpath-001)	III	Active, not recruiting 2023–203	Melanoma stages II–IV	Two groups: combination mRNA4157 + pembrolizumab, pembrolizumab monotherapy (control gro	No results posted
		NCT06077760 (INTerpath-002)	III	Open, recruiting 2023–2035	Resected non-small cell lung cancer, stages II, II		No results posted
Cevumeran (RO7198457)	USA, Memorial Sloan Kettering Cancer Center, Genen	NCT04161755	I	Active, not recruiting 2019–2025	Pancreatic cancer after surg	Sequential treatment: cevumeran, atezolizumab, followed by chemotherapy mFOLFIRINO	Treatment response was observed in 50% of patients (n = 8). Recurrence-free survival in 75% of responders (n = 6) exceeded 38 months. The median recurrence-free survival in non-responders (n = 8) was 13.4 month
	USA, BioNTech Ge	NCT03289962 (GO39733)	I	Active, not recruiting 2017–202	Locally advanced or metastatic solid tumors	Two groups: pembrolizumab monotherapy, combination mRNA-4157 + atezolizumab.	Neoantigen-specific T-cell responses were recorded in 71% of patients; 90% of patients receiving monotherapy and 92% receiving combination therapy experienced no adverse events of grade ≥3
		NCT03815058 (IMCODE00	II	Active, not recruiting 2019–2025	Metastatic or unresectable locally advanced melanoma stage IIIC/D	Two groups: combination Cevumeran + pembrolizumab, pembrolizumab monotherapy (control group).	No results posted
		NCT05968326 (IMCODE00	II	Open, recruiting 2023–2029	Ductal pancreas adenocarcinoma after surgical resection	Two groups: combination Cevumeran + pembrolizumab + chemotherapy mFOLFIRINOX, chemotherapy mFOLFIRINOX.	No results posted
		NCT06534983 (IMCODE004)	II	Open, recruiting 2024–2034	High-risk muscleinvasive urothelial carcinoma after surgical resection	Two groups: combination Cevumeran + nivolumab, nivolumab monotherapy.	No results posted
		NCT04486378 (BNT122-01)	II	pen, recruiting 2021–2030	Rectal cancer stage II/III or colon cancer stage II/III	Two groups: Cevumeran monotherapy, no treatment (watchful waiting).	No results posted
STZD-1801	China, Stemirna Therapeutics	NCT03908671	I	Open, recruiting 2019–2025	Advanced esophageal cancer (stage IIIc, IV) and non-small cell lung cancer (stages IIIB–IV)	mRNA vaccine monotherapy.	No results posted
SW1115C3		NCT05198752 (SWP1001-06)	I	Unknown status 2022–2024	Advanced solid tumors		All patients (n = 2) showed a treatment response. Recurrence-free survival was 8.4 months for the first patient and over 12 months for the second.
2020-06-mRNACOM		NCT05949775	Not Applicable	Active, not recruiting 2023–2026	Advanced solid tumors	Combination mRNA vaccine + sintilimab.	No results posted
2021-10-mRNACOM		NCT05761717	Not Applicable	Active, not recruiting 2023–2025	Hepatocellular carcinoma with postoperative recurrence	Combination mRNA vaccine + sintilimab.	No results posted
PGV002	China, NeoCura	NCT05192460 (XKY-1005)	Early I		Advanced gastric, esophageal, or liver cancer	Two groups: mRNA vaccine monotherapy, combination mRNA vaccine + PD-1/L1 antibody.	No results posted
Personalized neoantigen tumor vaccine		NCT05359354 (XKY-1007)	Not Applicable		Advanced solid tumors		No results posted
iNeo-Vac-R01	China, Hangzhou Neoantigen Therapeutics Co., Ltd.	NCT06019702 (SRRSH2023-755-01)	I	Open, recruiting 2023–2027	Advanced malignancies of the gastrointestinal system	mRNA vaccine monotherapy	No results posted
		NCT06026800(SRRSH2023-755-02)				First-line standard therapy, followed by mRNA vaccine monotherapy.	
		NCT06026774 (SRRSH2023-755-03)				Combination mRNA vaccine + standard adjuvant therapy.	No results posted
EVM16	China, Everest Medicines	NCT06541639 (EVM16CX01)	I	Open, recruiting 2023–2027	Any advanced or recurrent solid tumors	Two groups: mRNA vaccine monotherapy, combination mRNA vaccine + tislelizumab.	No results posted
SJ-Neo006	China, Jiangsu Synthgene Biotechnology	NCT06326736	Early I	Open, recruiting 2024-2026	Resectable ductal adenocarcinoma of the pancreas	Combination mRNA vaccine + camrelizumab + chemotherapy.	No results posted
mRNA tumor vaccines	China, Shanghai Regenelead Therapies	NCT06156267	Early I	Active, not recruiting 2024–2027	Adenocarcinoma of the pancreas after surgical resection	Combination mRNA vaccine + adebrelimab + mFOLFIRINOX chemotherapy.	No results posted
		NCT06735508 (NSCLC-IITRGL)	Early I	Active, not recruiting 2025–2026	Non-small cell lung cancer after surgical resection	Combination mRNA vaccine + adebrelimab.	No results posted
XP-004 Personalized mRNA Tumor Vaccine	China, Shanghai Xinpu BioTechnology Company Limited	NCT06496373 (2023PCV004)	I	Open, recruiting 2024–2027	Recurrent pancreatic cancer	Combination mRNA vaccine + PD-1 antibody.	No results posted

Personalized mRNA-based vaccines are currently used in combination with immune checkpoint inhibitors (ICIs) in clinical trials, as they help activated T-lymphocytes recognize tumor cell neoantigens and implement a full anti-tumor immune response. A class of preparations known as ICIs, which is quite common in oncology practice, includes monoclonal antibodies against cytotoxic T-lymphocyte antigen-4 (CTLA-4), a programmed cell death receptor (PD-1) and its ligand (PD-L1) (Table 2). CTLA-4, PD-1, and PD-L1 are surface receptors on T-cells that are necessary for their negative regulation [106]. Tumor cells use these molecules to deplete T-cells and “escape” the immune response. ICIs are designed to block this mechanism and restore the immune response suppressed by the tumor [107, 108].

**Table 2:** Immune checkpoint inhibitors used in clinical practice

Molecule targeted by the preparation	International name of the preparation	Trade name of the preparation	Oncologic ailments
CTLA-4	Ipilimumab	Yervoy	Unresectable or metastatic melanoma, renal cell carcinoma, colorectal cancer, hepatocellular carcinoma, non-small cell lung cancer
	Tremelimumab	Imjudo	
	Nurulimab	Nurdati	
PD-1	Prolgolimab	Forteca	Melanoma, non-small cell lung cancer, pancreatic cancer, oesophageal cancer, gastric cancer, breast cancer, prostate cancer, head and neck tumors, ovarian cancer
	Pembrolizumab	Keytruda Pembroria	
	Nivolumab	Opdivo	
	Camrelizumab	Areima	
PD-L1	Atezolizumab	Tecentriq	Bladder cancer, non-small cell lung cancer, breast cancer, hepatocellular carcinoma, metastatic melanoma, Merkel cell carcinoma, urothelial and renal cell carcinoma
	Avelumab	Bavencio	
	Durvalumab	Imfinzi	

ICIs monotherapy serves as a comparison group in studies of mRNA-based vaccines combined with ICIs (NCT03897881, NCT05933577, NCT03289962, NCT03815058), or mRNA vaccine monotherapy (NCT03289962, NCT05192460, NCT05359354, NCT06541639). A comparison of mRNA vaccine monotherapy with groups receiving standard conventional therapy appropriate for the selected cancer type is also available (NCT06295809, NCT04486378, NCT06026800). Expectations of potential success are high in combining ICIs with mRNA-based vaccines in clinical trials conducted on patients in the terminalstage of a disease for whom traditional treatments have proven ineffective (NCT03815058, NCT03289962, NCT 0 5 9 4 9 7 7 5 , NCT 0 5 1 9 2 4 6 0 , NCT 0 5 3 5 9 3 5 4 , NCT06541639).

**Clinical studies performed in the European Union and the United States**

*mRNA-4157 vaccine*. In 2017, Moderna (USA) initiated a Phase I clinical trial of the personalized mRNA-4157 vaccine (NCT03313778) (Fig. 2). In the first stage, patients with resected (part A) and unresectable (part B) solid tumors received four doses of mRNA-4157 monotherapy intramuscularly or combination therapy with pembrolizumab based on dose escalation regimens ranging from 0.04 to 1 mg. During the dose escalation stage, the group was divided into three parts: participants with unresectable, locally advanced or metastatic solid tumors (parts B and C) and resected cutaneous melanoma (part D). The patients were advised to use 1 mg of mRNA-4157, in combination with pembrolizumab and/or chemotherapy. In 2019, the first results were published, confirming the safety of the preparation by the absence of short-term severe adverse reactions (≥ grade 3) in all 33 patients, and its immunogenicity by the presence of multifunctional neoantigen-specific T-cells in response to target neoantigens in each patient. Among the 13 patients who received adjuvant monotherapy with mRNA4157, 92.3% showed no evidence of disease at a median follow-up time length of 8 months. The remaining 20 patients received combination therapy consisting of a mRNA vaccine and pembrolizumab, and 14 of them responded to combination therapy: in half of the cases, partial remission or stabilization of the disease was observed, while in the other half, disease progression or immunosuppression was observed. Consequently, mRNA-4157 proved safe and well tolerated at all tested dose levels. These results confirmed the efficacy of the target neoantigen selection algorithm and highlighted the promising clinical application of the personalized neoantigen mRNA vaccine therapy strategy, which made possible for mRNA-4157 to advance to Phase II clinical trials [109].

At the Society for Immunotherapy of Cancer conference (San Diego, California, USA) held in November 2023, the results obtained with mRNA-4157 use were supplemented: In evaluating safety and tolerability, all patients experienced ≥ 1 adverse event during treatment; no dose-limiting adverse events of grade 4 or 5 severity had been observed. The most common adverse events were fatigue (67%), fever (60%), and pain at the injection spot (40%). T-cell responses were observed in all patients, 85% of which were identified as de novo responses. The highest frequency of these responses was achieved after the beginning of combination therapy with pembrolizumab. It was also observed that a high percentage of immune responses to the combination of mRNA-4157 with pembrolizumab in patients was associated with an activated T-cell phenotype, while a low percentage was associated with the prevalence of a naive T-cell phenotype [89]. The mRNA-4157 study is ongoing and forms the basis for phases involving groups of patients with tumors in other locations.

In 2019, the KEYNOTE-942 (Phase II) study was initiated to evaluate the efficacy of the personalized mRNA-4157 vaccine in patients with stage IIIB-D and IV melanoma after complete surgical resection with a high risk of recurrence (NCT03897881). Patients received combination therapy with mRNA-4157 and pembrolizumab (n = 107) or pembrolizumab monotherapy (n = 50). The mRNA-4157 vaccine (1 mg) was administered intramuscularly nine times at threeweek intervals, and pembrolizumab (200 mg) was administered intravenously every three weeks for 18 cycles. With a minimum follow-up period of 14 months in the group of patients who completed the full course of treatment, adverse outcomes (relapse or death) were observed in 22% (24/107) of the patients in the combination therapy group and in 40% (20/50) of patients who received monotherapy. Recurrencefree survival was better in the combination therapy group than in the monotherapy group (83% versus 77% at 12 months and 79% versus 62% at 18 months). Cases of distant recurrence or death after 24 months were observed in 8% of patients after combination therapy and 24% of patients under monotherapy [110].

Encouraging results from Moderna and Merck & Co’s (USA) mRNA-4157 vaccine have resulted in the initiation of the studies NCT06307431 and NCT06305767, which began in 2024. It is anticipated that mRNA-4157 therapy in combination with pembrolizumab will be more efficient than pembrolizumab monotherapy in renal cell carcinoma (NCT06307431) and standard treatment in muscle-invasive urothelial carcinoma (NCT06305767). These trials cover between 8 and 15 countries and will continue until 2031–2032. In addition, in early 2025, Moderna, in partnership with Merck & Co (USA), initiated Phase III clinical trials of the mRNA-4157 vaccine, in combination with pembrolizumab, for the treatment of squamous cell skin carcinoma (NCT06295809), melanoma (NCT05933577), and non-small cell lung cancer (NCT06077760). Each study involves between 20 and 33 countries and between 868 and 1,089 patients.

*Cevumeran*. In 2017, BioNTech (Germany) and Genentech (USA) initiated Phase I clinical trials of the mRNA preparation Cevumeran (NCT03289962) intended for the treatment of patients with melanoma, head and neck cancer, colorectal cancer, non-small cell lung cancer, bladder cancer, and other progressive solid tumors (Fig. 2). The safety, immunogenicity, and preliminary efficacy of monotherapy (n = 30) and in combination with atezolizumab (n = 183) were evaluated in patients who had received prior therapy. According to safety data, 9 out of 30 patients receiving Cevumeran monotherapy and 47 out of 183 patients receiving combination therapy with atezolizumab discontinued treatment as a result of ailment expansion. Side effects were noted in 90% of the patients receiving Cevumeran monotherapy: in 3 patients, they were classified as grade 3, and in the remaining 24 patients they were classified as grade 1 or 2. One case of dose-limiting toxicity grade 3 was observed during monotherapy with 100 μg of Cevumeran, but after the side effects had dissipated, the patient continued to participate in the study at a reduced dose until ailment progression on day 82. Three grade 4 or 5 adverse events were recorded during the combined use of Cevumeran and atezolizumab; subsequently, 11 patients discontinued treatment as a result of adverse immune-mediated reactions, predominantly in the atezolizumab monotherapy group. The remaining participants experienced minimal side effects, the most common of which were infusion reactions (56.7% and 59.6% for monotherapy and combination therapy, respectively), cytokine release syndrome (30% and 20.8%), and flulike symptoms (3.3% and 12.6%). In the preliminary efficacy assessment, 71% of the patients demonstrated a polyepitope neoantigen-specific response involving CD4^+^ and/or CD8^+^ T-cells, which persisted for up to 23 months. At the same time, CD8^+^ T-cells specific to several neoantigens constituted an average of 7.3% of the circulating pool of CD8^+^ T-cells and were also detected in tumor foci, comprising up to 7.2% of the total number of tumor-infiltrating T-cells. No statistically significant results about a correlation between the clinical effect and immune response were obtained due to the limited volume and heterogeneity of the samples for each tumor type. A patient with microsatellite-stable rectal cancer (low PD-L1 expression) demonstrated a complete response to combination therapy with autologous Cevumeran (9 doses of 38 mcg) and atezolizumab for 8.2 months. A patient with highly differentiated breast cancer (high PD-L1 level) due to tumor progression against a background of experimental treatment with nivolumab was transferred to autologous Cevumeran at a dose of 38 mcg and atezolizumab, which led to a partial response with a reduction in the size of metastases in the lungs over a period of 9.9 months. These results justified further study of Cevumeran and became the basis for new phase I–II clinical trials [111].

In 2019, BioNTech (Germany) and Genentech (USA) jointly initiated Phase I clinical trials of Cevumeran against resected pancreatic adenocarcinoma (NCT04161755). The study included 16 patients who received atezolizumab and Cevumeran after surgery, 15 of whom then underwent chemotherapy with mFOLFIRINOX. The safety profile was assessed based on the number and severity of adverse reactions, and preliminary efficacy was assessed based on T-cell specificity to the vaccine neoantigens, recurrence-free survival, and overall survival at 18 months. In 15 of the 16 patients, autologous Cevumeran was tolerated without grade 3–5 adverse reactions; one patient experienced fever and hypertension, which were assessed as a grade 3 adverse reaction. The appearance of neoantigen-specific T cells was noted in 8 out of the 16 patients, accounting for up to 10% of all blood T-cells The cells retained functionality and produced IFN-γ. Even after chemotherapy and were reactivated upon administration of a booster dose of the vaccine. They also included up to 2.5% of multifunctional neoantigen-specific effector CD8^+^ T-cells that persisted for 2 years after the surgery. During 18 months of follow-up, the median overall and recurrence-free survival in eight patients with a T-cell response to the vaccine exceeded 18 months, while in eight non-responders, the median recurrence-free survival time was 13.4 months. Since T-cell activity in patients with resected pancreatic adenocarcinoma correlated with delayed recurrence, a global randomized phase II trial was initiated [30].

Although initial results were published in 2023, long-term follow-up of Phase I participants remained ongoing, leading to updated findings from the NCT04161755 trial in 2025. With a median follow-up period of 3.2 years (2.3–4.0 years), all eight patients who responded to therapy remained recurrence-free. Consequently, six out of eight respondents remained in remission, while seven out of eight who did not respond to therapy experienced a relapse. Additionally, the origin and lifespan of specific T-cell clones were studied. It has been revealed that Cevumeran induces CD8^+^ T-cell clones with an average lifespan of 7 years. At the same time, vaccine-induced clones are not observed in tissues prior to vaccination, and 86% of them retain the cytotoxic, tissue-resident state of memory T-cells for 3 years after vaccination, while preserving neoantigen-specific effector function. This observation led to the conclusion that there is a consistent correlation between the response to the vaccine and progression-free survival for 3.6 years [32].

In 2023, a randomized phase II trial of the preparation Cevumeran (NCT05968326) was initiated. The treatment regimen for patients with pancreatic ductal adenocarcinoma following surgical resection included a combination of Cevumeran, atezolizumab, and mFOLFIRINOX chemotherapy versus single-agent chemotherapy. In addition, as part of Phase II trials, the preparation Cevumeran, in combination with ICIs, was being administered to patients with melanoma (NCT03815058) and muscle-invasive urothelial carcinoma (NCT06534983), and as monotherapy to patients with rectal or colon cancer (NCT04486378). The NCT03815058 study was completed in January 2025, but the results are not yet available. The completion of the other studies should not be expected before 2029.

**Clinical studies performed in China**

A total of 14 early Phase I and II trials listed on clinicaltrials.gov are underway in China as of January 2025, and five in the United States (Table 1). Chinese companies such as Stemirna Therapeutics, NeoCura, Everest Medicines, Hangzhou Neoantigen Therapeutics, Jiangsu Synthgene Biotechnology, Shanghai Regenelead Therapies, and Shanghai Xinpu BioTechnology are active in the development of mRNA-based vaccines.

In October 2024, the results of preclinical studies of the Chinese anti-tumor vaccine SW1115C3 [112] were published. The preparation proved to be efficient in mouse models of CT26, MC38, and B16F10 tumors by activating neoantigen-specific cytotoxic T-cells and inducing the secretion of cytotoxic cytokines. This encouraged the move to Phase I clinical trials on two patients. The first patient with advanced stomach cancer, multifocal metastases, and a low mutation burden, achieved a recurrence-free survival period of 8.4 months and partial remission after receiving a combination of SW1115C3 with vedolizumab and pembrolizumab. The second patient with type B luminal breast cancer after neoadjuvant therapy and mRNA vaccine treatment evinced a persistent T-cell response to 11 out of 20 neoantigens. One year after surgery, she shows no evidence of recurrence or metastasis, and monitoring continues.

Stemirna Therapeutics has initiated studies to evaluate the efficacy of STZD-1801 monotherapy in patients with esophageal cancer and non-small cell lung cancer (NCT03908671), as well as combination therapy with a mRNA-based vaccine and stintilumab for advanced solid tumors (NCT05949775) and hepatocellular carcinoma (NCT05761717) [80]. NeoCura has initiated a study of the efficacy of mRNA-based monotherapy and combination therapy with ICIs in patients with advanced solid tumors (NCT05359354), with a separate study focusing on patients with gastric, esophageal, or liver cancer (NCT05192460). The anti-tumor mRNA-based vaccine iNeo-Vac-R01 from Hangzhou Neoantigen Therapeutics (China) targets common neoplasms of the digestive system and is being studied in three parallel trials to select the most effective treatment strategy (NCT06019702, NCT06026800, NCT06026774). The EVM16 mRNAbased vaccine from Everest Medicines is in Phase I clinical trials, including patients with recurrent or advanced solid tumors receiving monotherapy with the vaccine or combination therapy with tislelizumab (NCT06541639). The efficacy of various combinations of anti-tumor mRNA-based vaccines with chemotherapy and ICIs is being evaluated in studies initiated by Jiangsu Synthgene Biotechnology, Shanghai Regenelead Therapies, and Shanghai Xinpu BioTechnology Company Limited. The study groups include patients with pancreatic cancer (NCT06326736, NCT06156267, NCT06496373) and non-small cell lung cancer (NCT06735508). The results of all these studies, however, have not yet been published.

**Studies performed in the Russian Federation**

In September 2024, the National Research Center for Epidemiology and Microbiology named after Honorary Academician N.F. Gamaleya of the Ministry of Health of the Russian Federation announced the completion of preclinical trials of a domestic mRNA-based vaccine against melanoma, developed jointly with the National Medical Research Radiological Centre of the Ministry of Health of the Russian Federation1 (Fig. 2). According to the official website of the N.N. Blokhin National Medical Research Centre of Oncology, patient enrollment for Phase I clinical trials is not expected until the second half of 2025.

A scientific and technological center for the development of mRNA technologies has been established in accordance with Decree No. 195-r of the Government of the Russian Federation dated February 3, 2025. The functions of this leading scientific organization are entrusted to the Federal State Budgetary Institution “National Research Center for Epidemiology and Microbiology named after Honorary Academician N.F. Gamaleya” of the Ministry of Health of the Russian Federation.

## CONCLUSION

mRNA-based vaccines designed to encode defined tumor antigens have shown robust clinical activity, either alone or in synergy with immune checkpoint inhibitors (ICIs), in multiple oncological indications. The platform’s versatility, embodied in broad target selection (notably neoantigens), tunable mRNA constructs, and interchangeable delivery systems, points to its capacity for rapid development and implementation in real-world oncology settings.

## Abbreviations

APCs: antigen-presenting cells
LNPs: lipid nanoparticles
LPPs: lipopolyplexes
PEG: polyethylene glycol
CpG-ODNs: CpG-oligodeoxynucleotides
BDMPs: biotechnology-derived medicinal preparations
CTLA-4: cytotoxic T-lymphocyte antigen-4
PD-1: programmed cell death receptor
PD-L1: programmed cell death receptor ligand
